# Impact of redox-related genes on tumor microenvironment immune characteristics and prognosis of high-grade gliomas

**DOI:** 10.3389/fncel.2023.1155982

**Published:** 2023-05-12

**Authors:** Yunbo Yuan, Mingrong Zuo, Shuxin Zhang, Siliang Chen, Wentao Feng, Zhihao Wang, Mina Chen, Yanhui Liu

**Affiliations:** ^1^Department of Neurosurgery, West China Hospital, Sichuan University, Chengdu, China; ^2^Department of Biotherapy, Cancer Center and State Key Laboratory of Biotherapy, West China Hospital, Sichuan University, Chengdu, China

**Keywords:** glioma, immune, prognosis, redox, microenvironment

## Abstract

**Introduction:**

High-grade glioma (HGG) defines a group of brain gliomas characterized by contrast enhancement, high tumor heterogeneity, and poor clinical outcome. Disturbed reduction–oxidation (redox) balance has been frequently associated with the development of tumor cells and their microenvironment (TME).

**Methods:**

To study the influence of redox balance on HGGs and their microenvironment, we collected mRNA-sequencing and clinical data of HGG patients from TCGA and CGGA databases and our own cohort. Redox-related genes (ROGs) were defined as genes in the MSigDB pathways with keyword “redox” that were differentially expressed between HGGs and normal brain samples. Unsupervised clustering analysis was used to discover ROG expression clusters. Over-representation analysis (ORA), gene set enrichment analysis (GSEA) and gene set variation analysis (GSVA) were also employed to understand the biological implication of differentially expressed genes between HGG clusters. CIBERSORTx and ESTIMATE were used to profile the immune TME landscapes of tumors, and TIDE was used to evaluated the potential response to immune checkpoint inhibitors. Least Absolute Shrinkage and Selection Operator (LASSO) Cox regression was used to construct HGG-ROG expression risk signature (GRORS).

**Results:**

Seventy-five ROGs were found and consensus clustering using the expression profile of ROGs divided the both IDH-mutant (IDHmut) and IDH-wildtype (IDHwt) HGGs into subclusters with different prognosis. Functional enrichment analysis revealed that the differential aggressiveness between redox subclusters in IDHmut HGGs were significantly associated with cell cycle regulation pathways, while IDHwt HGG redox subclusters showed differentially activated immune-related pathways. *In silico* TME analysis on immune landscapes in the TME showed that the more aggressive redox subclusters in both IDHmut and IDHwt HGGs may harbor a more diverse composition of tumor-infiltrating immune cells, expressed a higher level of immune checkpoints and were more likely to respond to immune checkpoint blockade. Next, we established a GRORS which showed AUCs of 0.787, 0.884, and 0.917 in predicting 1–3-year survival of HGG patients in the held-out validation datasets, and the C-index of a nomogram combining the GRORS and other prognostic information reached 0.835.

**Conclusion:**

Briefly, our results suggest that the expression pattern of ROGs was closely associated with the prognosis as well as the TME immune profile of HGGs, and may serve as a potential indicator for their response to immunotherapies.

## Introduction

Glioma is the most common nervous malignancy in the adult population (Ostrom et al., 2021). High-grade gliomas (HGGs) refer to grade 3 and grade 4 gliomas (Louis et al., 2016, 2021). Pathologically, HGGs are characterized by large fraction of proliferating tumor cells, immune cell infiltration, marked angiogenesis and necrosis (Ertosun and Rubin, 2015). Compared to less aggressive grade 1 and grade 2 gliomas, HGGs exhibit remarkable inter- and intra-tumoral heterogeneity, and poorer clinical outcomes (OS) (Nakamura and Takada, 2021; Sun and Kim, 2022). Despite phase III trials revealing that non-specific eradicating proliferating malignant cells, including radiation, chemotherapy, and tumor-treating fields, confers survival benefits for gliomas, their curative effect on HGGs remains modest (Chen et al., 2017). Therefore, the in-depth mechanisms underlying the malignant progression of tumors should be explored to help seek novel therapeutic targets for HGGs more precisely.

Redox balance is defined as the dynamic balance between reactive oxygen species (ROS), reactive nitrogen species (RNS), and anti-oxidants (Chaiswing and Oberley, 2010). Redox stress involves in varied pathophysiological processes; for example, ferroptosis, a recently identified form of programmed cell death, is triggered by lipid peroxidation (Hassannia et al., 2019). Excessive ROS can be observed in a variety of diseases, including inflammatory diseases, cardiovascular diseases and malignant cancers (Gorrini et al., 2013; Madreiter-Sokolowski et al., 2020; Mullen et al., 2020). Redox stress could regulate proliferation, invasion, metastasis, drug resistance and tumor microenvironment (TME), and redox system has been exploited as a potential target for cancer treatment (Pitt et al., 2016; Lee et al., 2017; Serrano et al., 2020; Nakamura and Takada, 2021).

Almost all cells in the TME produce ROS which could activate or inhibit the infiltrating immune cells and regulate tumor cell progression (Hegedus et al., 2018). A study has reported that different redox gene status is associated with biological features in prostate cancers, for example, the immune condition (Wu Y. et al., 2021). In gliomas, overexpression of Nrf2 and knockdown of Keap1 could promote proliferation by promoting xCT function, which could switch redox status (Fan et al., 2017). Although Redox can regulate multiple malignant characteristics of tumors, genetic evidence dissecting the molecular mechanisms by which redox-related genes (ROGs) regulate the malignant progression of HGGs and the prognostic value of ROGs are still required.

In this study by analyzing data based on HGG cohorts from public databases, we found 75 glioma-specific ROGs and divided the HGG patients into distinct groups based on their expression patterns. We also established a scoring system based on ROGs and a nomogram to estimate the relative risk levels of HGG patients and subsequently predict their clinical outcomes. Our established expression pattern of ROGs were closely associated with the prognosis as well as the TME immune profile of HGGs, and may serve as a potential indicator for their response to immunotherapies.

## Materials and methods

### Data sources

Transcription data and the corresponding clinical data were downloaded from The Cancer Genome Atlas (TCGA)^1^ database and dataset mRNAseq_325 in Chinese Glioma Genome Atlas (CGGA)^2^ database (Zhao et al., 2021). HGG patients were defined as those graded as WHO grade 3 or 4 histologically. Although IDH-wildtype diffuse gliomas with WHO grade 2 morphology features were now classified as grade 4 glioblastomas thus HGG in the most recent version of WHO classification system, we proceeded the study by only taking those already classified as HGG in their original clinical information curated in the repositories, so that that the definition of HGG were consistent regardless of different classification guideline version (Louis et al., 2016, 2021). This study only included adult primary HGG patients with complete survival data. 391 patients from TCGA database and 132 patients from CGGA database (dataset mRNAseq_325) were included. 48 HGG patients from West China Hospital (WCH) were also included with the same criteria. The RNA-seq data were from the samples of these patients’ tumor tissue obtained during craniotomy, and then quantified with STAR. The follow-up period was set as 3–6 months after the patient received surgical intervention. These patients formed three cohorts according to the data sources: TCGA cohort, CGGA cohort, and WCH cohort. The data of gene expression was presented as fragments per kilobase million (FPKM) if not otherwise stated. And detailed information of the patients is presented in Supplementary Table 1.

### Screening of redox-related genes

To figure out differentially expressed genes (DEGs) between glioma samples and normal brain samples, we filter genes with criteria of a significant difference (adjusted *P*-value < 0.05) and an expression level that was not excessively low (maximum FPKM > 0.1) with the R package “limma.” The ROGs were retrieved from the Molecular Signature Database (MsigDB, v7.5.1)^3^ (Subramanian et al., 2005; Liberzon et al., 2011) with the keyword “redox” (Supplementary Table 2). And the overlapped genes between DEGs and ROGs, which were supposed to be “differentially expressed ROGs,” would be included for further analyses.

### Unsupervised clustering analysis

For exploration of different mode of redox gene expression, R package “ConsensusClusterPlus” was employed for unsupervised consensus clustering analysis to classify redox patterns in terms of the expression levels of ROGs (Wilkerson and Hayes, 2010). Considering the impact of IDH mutation status on the prognosis of HGG patients (Yan et al., 2009), this clustering was also applied to different IDH subgroups after stratification. The clustering was supposed to follow those criteria: a sufficient sample size and a gradually increased smooth cumulative distribution function (CDF) curve, and thus the number and components of clusters were determined. Subsequently, principal component analysis (PCA) of these included ROGs was applied to visualize the differences of patterns of redox gene expression patterns between clusters.

### Functional gene enrichment analyses

Gene enrichment analyses were conducted to explore potential mechanisms based on DEGs from different subgroups (including consensus clusters and risk groups identified in following analyses, respectively). With Kyoto Encyclopedia of Genes and Genomes (KEGG), reactome gene sets (REACTOME) from MSigDB and gene ontology (GO) enrichment, over-representation analysis (ORA) and gene set enrichment analysis (GSEA) were performed to explore in which biological processes these DEGs were enriched using the R package “clusterProfiler” (Yu et al., 2012; Wu T. et al., 2021). Gene set variation analysis (GSVA) was also employed to generate the pathway expression matrix of genes from the logFPKM matrix using “GSVA” package in R, so as to figure out the differences in signaling pathways between groups (Hanzelmann et al., 2013). The processes and pathways identified above would be seen as associated with the ROG patterns (or risk groups).

The clinical characteristics of each subgroup were analyzed with student *t*-test or chi-square test according to the statistical type of data (chi-square test for discrete variables and student *t*-test for continuous variables) to explore the relation between ROG patterns (or risk groups) and clinical features. The characteristics included age, gender, WHO grade, IDH status, 1p19q codeletion, ATRX status, MGMT promoter status, and TERT promoter status.

### Analyses on tumor microenvironment immune characteristics

To study the TME and immune state of each HGG sample, A set of analyses on TME immune features were performed. CIBERSORTx algorithm,^4^ whose assessment is based on a validated reference gene signature matrix, was used for calculation of the proportion of each infiltrating immune cells (Newman et al., 2015), while Estimation of Stromal and Immune cells in Malignant Tumor tissues using Expression data (ESTIMATE) score were utilized to examine the differences of tumor stromal and immune microenvironment (Yoshihara et al., 2013). And ESTIMATE tumor purity and consensus purity estimation (CPE) data were applied to assess tumor purity of those glioma samples by calculating the stromal, immune and ESTIMATE scores (ESTIMATE tumor purity) or obtaining the median purity from different purity-estimating methods including ESTIMATE after normalization (CPE), respectively, (Yoshihara et al., 2013; Aran et al., 2015). The analyses above could unveil the estimated cellular and microenvironmental characteristics of HGGs. We also employed the Tumor Immune Dysfunction and Exclusion (TIDE) algorithm for the data of T-cell dysfunction and exclusion, hence to predict the therapeutic response of immune checkpoint blockades (ICBs) in treating HGGs (Jiang et al., 2018).

### Calculation of glioma-redox risk score and nomogram establishment

To establish a prognostic model for HGG patients, patients in TCGA cohort were first randomly divided into two cohorts, the TCGA training cohort and the TCGA validation cohort, with a ratio of 6:4. Next, the DEGs were first filtered by the Least Absolute Shrinkage and Selection Operator (LASSO) Cox regression with the criteria of adjusted *P*-value < 0.05 based on TCGA training cohort. LASSO Cox regression was performed with the package “glmnet” in R and repeated 100 times (Friedman et al., 2010). The genes with coefficient that was not 0 at the lambda minimum concordance index (C-index) would be regarded as prognostically relevant. And then based on expression levels of these selected genes, a score for prognosis prediction called HGG-ROG expression risk signature (GRORS) would be calculated as the following formula displays:


$$G\,R\,O\,R\,S=\sum c\,o\,e\,f_{i}\times F\,P\,K\,M_{i}$$

The optimal cutoff of GRORS was calculated by “survminer” R package, which divided the HGG patients into two groups, high-risk group (GRORS ≥ optimal cutoff) and low-risk group (GRORS < optimal cutoff). And univariate Cox regression and multivariate Cox regression were conducted to screening independent prognostic factors, which could also test whether GRORS was one of them. Next, with the help of the package “timeROC” in R, the AUC (area under the curve) of ROC (receiver operating characteristic) curve was evaluated at the timepoint of 1–3 year (s) after diagnosis, so as to assess the prognostic-predicting potential of GRORS (Blanche et al., 2013). Subsequently a nomogram was established with the selected independent prognostic factors with the R package “rms,” and its accuracy was assessed by the calibration curve and corrected C-index. The validation of the nomogram would be conducted with TCGA validation cohort, CGGA cohort, and WCH cohort.

### Statistical analyses

R interpreter (version 3.6.1) and the above-mentioned R package were used to handle the RNA-sequencing relevant data. Kaplan–Meier (K-M) analysis was conducted to evaluate prognosis of specific glioma groups using log-rank test. A two-sided *p* < 0.05 was regarded as statistically significant and * indicated *p* < 0.05, whereas ^**^p < 0.01, ^***^p < 0.001 and ^****^p < 0.0001 in this study.

## Results

### Identification of DEGs and ROGs in gliomas and clustering of patients

**FIGURE 1:**
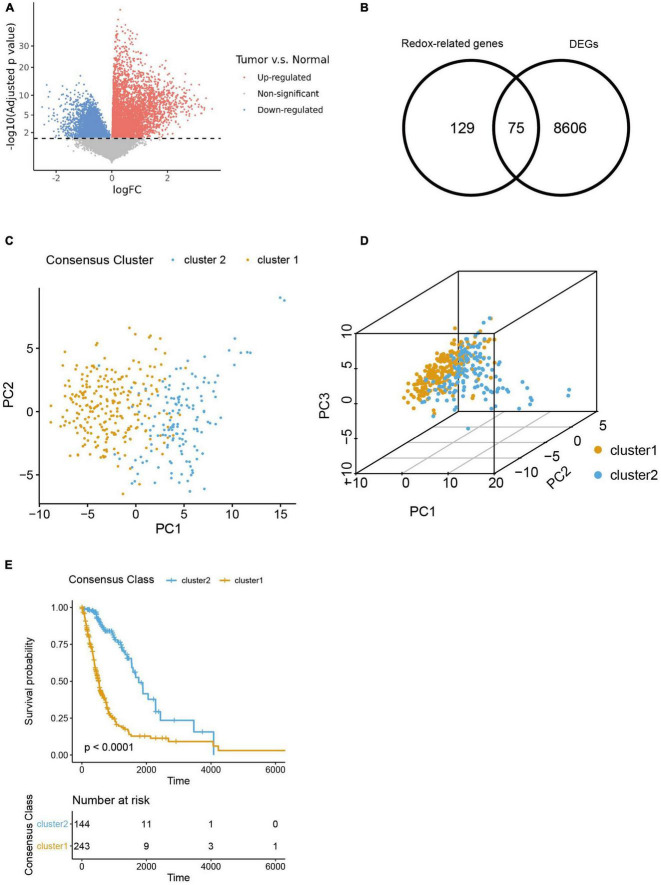
Selection of redox-related genes (ROGs). **(A)** Volcano plot of gene fold change between glioma samples and normal brain samples from TCGA sets. **(B)** Venn diagram showing intersection of DEGs and ROGs. **(C,D)** 2- and 3-dimensional PCA for two consensus clusters based on ROGs. **(E)** K-M curves of the consensus clusters.

We firstly figured out 8,606 DEGs by analyzing the differences in transcriptomic expression between samples of HGG patients (*n* = 391) and normal brain (*n* = 5) from TGCA databases (Figures 1A, B). And we identified 129 ROGs by retrieving the MSigDB database with the keyword “redox.” Then 75 genes were extracted as the intersection of the DEG and ROG sets as portrayed in the Venn Diagram (Figure 1B). Subsequently, patients from the TCGA database were divided into two consensus clusters with a method of consensus clustering according to the transcription profiles of these ROGs. As shown in the PCA plots (Figures 1C, D), there are significant differences between the two clusters, affirming the validity of this clustering. We next assessed the OS for the two clusters, and observed significantly worse survival of patients of cluster 1 was found as compared to their counterpart (Figure 1E).

**FIGURE 2:**
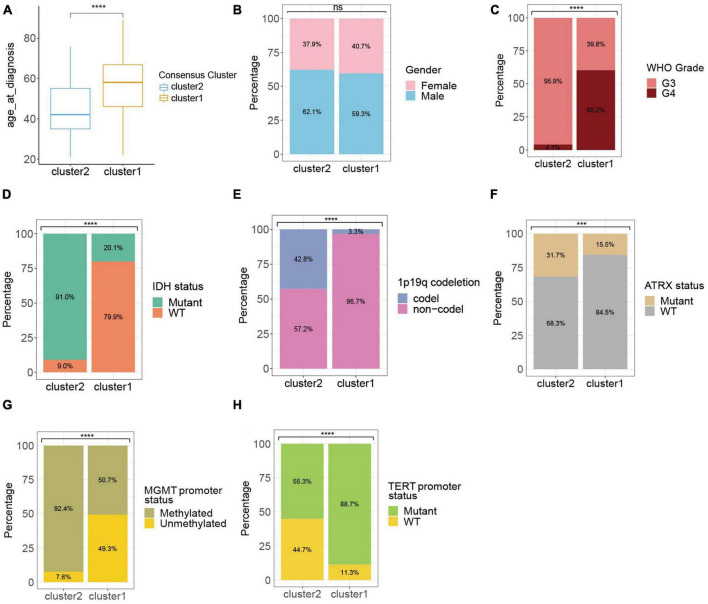
Clinic-pathological characteristics of the ROG clusters from the TCGA cohort. Compared characteristics of clusters included age at diagnosis **(A)**, gender **(B)**, WHO grade **(C)**, IDH status **(D)**, 1p19q codeletion **(E)**, ATRX status **(F)**, MGMT promoter status **(G)**, and TERT promoter status **(H)**. ns, not significant; ***p < 0.001, ****p < 0.0001.

Considering the significant difference in the survival outcome between gliomas of different clinicopathological profiles, we next explored the associations between ROG expression patterns in HGGs and their clinical characteristics. Results showed that comparing to cluster 1, the HGG patients of cluster 2 had a younger age, lower WHO grades, higher proportion of IDH mutation, 1p19q codeletion, ATRX mutation and MGMT promoter methylation, and less likelihood of TERT promoter mutation (Figures 2A–H).

### Redox subclusters in IDH mutant and wildtype HGGs

**FIGURE 3:**
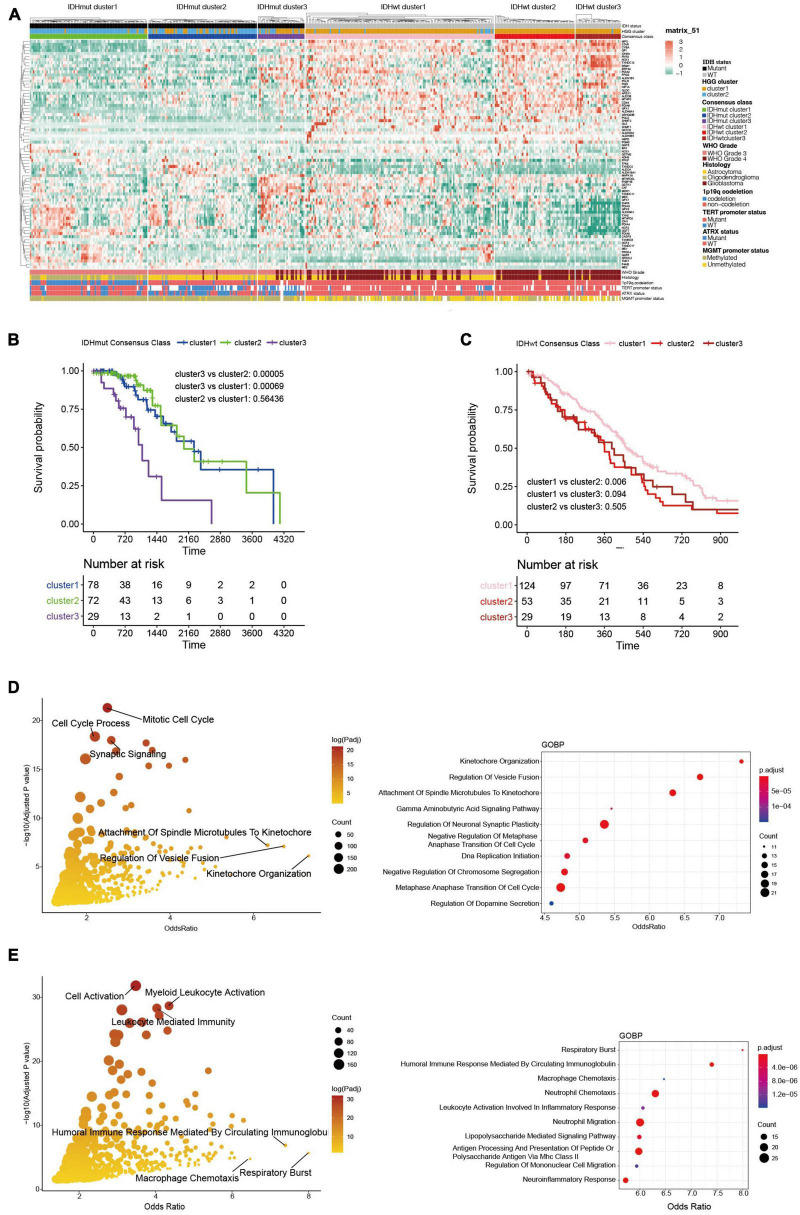
Transcriptome and clinicopathological profiles of redox subclusters in IDH-mutant (IDHmut) and IDH-wildtype (IDHwt) HGGs, respectively. **(A)** Heatmap of ROG expression in the subclusters. **(B)** K-M curves of IDHmut subclusters. **(C)** K-M curves of IDHwt subclusters. **(D)** Gene ontology: biological pathways (GOBP) functional enrichment of differentially expressed genes between subcluster 1/2 and subcluster 3 in the IDHmut HGGs; only pathways with gene counts over 10 were plotted; right panel enriched pathways with top 10 odds ratio. **(E)** Gene ontology: biological pathways (GOBP) functional enrichment of differentially expressed genes between subcluster 1 and subcluster 2/3 in the IDHwt HGGs. Right panel enriched pathways with top 10 odds ratio.

The WHO grade and IDH mutational status are known to significantly influence the prognosis of HGGs (Yan et al., 2009; Chen et al., 2017). Particularly, IDH-wildtype gliomas (IDHwt) generally had worse survival outcome compared to IDH-mutants despite their WHO grades (Eckel-Passow et al., 2015). Therefore, we further explored the heterogeneity of ROG expression after stratifying the HGGs into IDH-mutants (IDHmut) and IDHwt. Consensus clustering divided both IDHmut and IDHwt HGGs into three subclusters (Figure 3A and Supplementary Figures 1A–F). In IDHmut HGGs, cluster 3 had significantly worse survival outcome than the other two clusters (Figure 3B). In IDHwt HGGs, cluster 1 showed significantly better prognosis than cluster 2 and near-significantly better prognosis than cluster 3 (Figure 3C). To understand potential mechanism behind the aggressiveness differences between the subclusters, we analyzed the functional enrichment of differentially expressed genes (DEGs) between IDHmut cluster3 and cluster 1/2, as well as those between IDHwt cluster 1 and cluster 2/3. In IDHmut HGGs, the DEGs were significantly enriched in cell cycle regulation and neuronal functions (Figure 3D and Supplementary Figures 2A–C), while in IDHwt HGGs, the DEGs were significantly enriched in respiratory burst and immune-related pathways (Figure 3E and Supplementary Figures 3A–C). These findings suggest that the ROG expression patterns drove aggressiveness in IDHmut and IDHwt HGGs through different mechanisms.

### Immune phenotypes of IDH mutant and wildtype HGG subclusters

**FIGURE 4:**
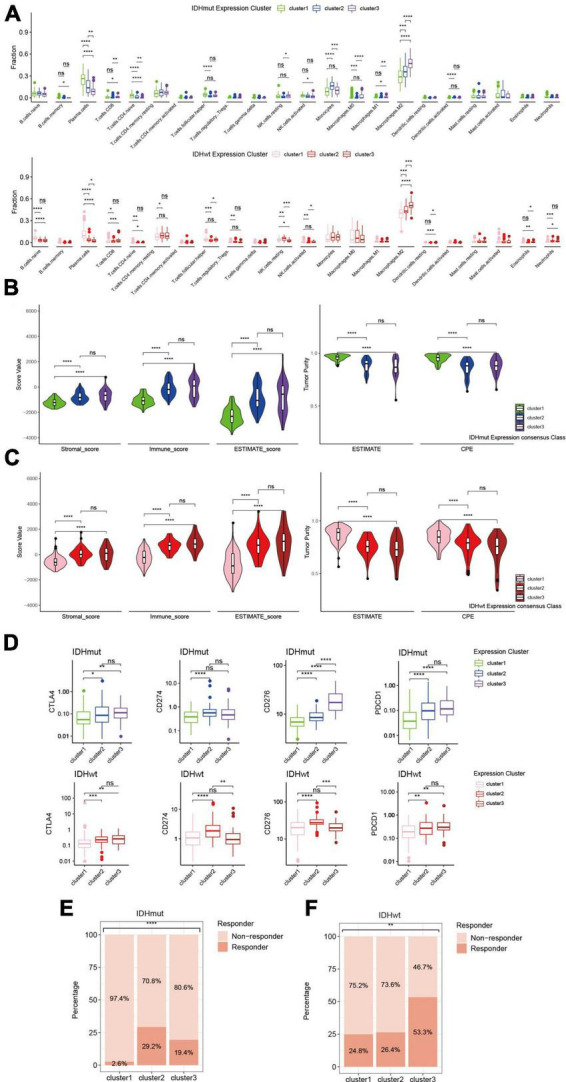
Immune phenotypes of redox subclusters in IDH-mutant (IDHmut) and IDH-wildtype (IDHwt) HGGs. **(A)** Fraction of 22 tumor infiltrating immune cells computed with the CIBERSORTx algorithm. **(B)** Stromal, immune and ESTIMATE score with the method of ESTIMATE. **(C)** Tumor purity estimation with the ESTIMATE and consensus purity estimation (CPE). **(D)** mRNA expression of CD274 (PD-L1), PDCD1 (PD1), CTLA4, and CD276 (B7-H3). **(E,F)** Percentage of estimated responders to ICBs in IDHmut and IDHwt subclusters with TIDE. ns, not significant; *p < 0.05, **p < 0.01, ***p < 0.001, ****p < 0.0001.

Since immune-related pathways were implicated in the aggressiveness difference between redox subclusters in both IDHmut and IDHwt HGGs, we then investigated their immune profiles with a series of algorithms that dissect the immune TME of HGGs *in silico* based on their transcriptome. CIBERSORTx analysis found significantly lower infiltration of plasma cells and higher infiltration of M2 macrophages in the more aggressive redox subclusters for both IDHmut and IDHwt HGGs (Figure 4A). Consistently, in the ESTIMATE analysis, cluster 1 in both IDHmut and IDHwt had significantly lower stromal and immune cell infiltration and higher tumor purity compared to the other two redox subclusters (Figures 4B, C). Meanwhile, expression of immune checkpoints, including CD274 (PD-L1), PDCD1 (PD1), CTLA4, and CD276 (B7-H3), were generally higher in cluster 2/3 of both IDHmut and IDHwt HGGs (Figure 4D and Supplementary Figure 4). TIDE analysis found lower fraction of potential ICB responders in the cluster 1 of IDHmut HGGs than the other two redox subclusters (Figure 4E). In IDHwt HGGs, cluster 3 had higher proportion of potential ICB responders compared to the other two redox subclusters (Figure 4F). These results suggest that the expression pattern of ROGs had significant impact on the immune TME of both IDHmut and IDHwt HGGs.

### HGG-ROG expression risk signature (GRORS) and its prognostic value

**FIGURE 5:**
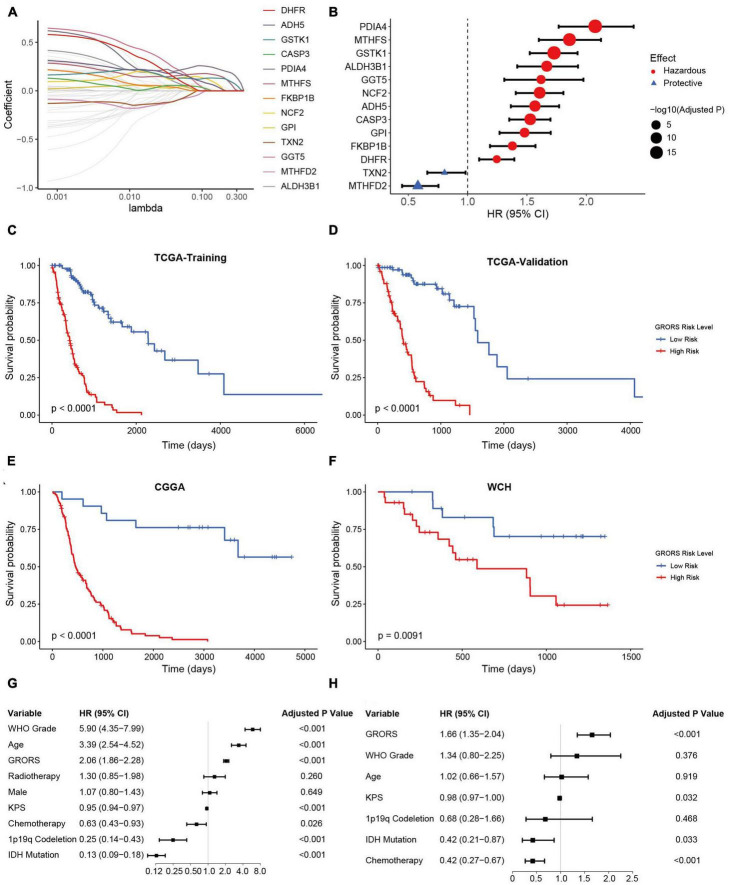
Development and validation of prognostic GRORS. **(A)** Average of coefficients of each ROG in the LASSO Cox regression model at different lambda values. **(B)** Forest plots showing HRs of each selected ROG. **(C–F)** K-M curves for high- and low-risk group divided by GRORS in TCGA training cohort **(C)**, TCGA validation cohort **(D)**, CGGA cohort **(E)**, and WCH cohort **(F)**. **(G,H)** Forest plots showing the HRs of GRORS and clinicopathological indicators in univariate Cox regression **(G)** and multivariate Cox regression **(H)**.

The influences of ROG expression pattern on both IDHmut and IDHwt HGGs suggest that a unified prognosis prediction score could be established using ROG expression. To set up the scoring system for assessing the prognosis of HGG patients, 13 ROGs were found to be independent prognostic factors calculated by LASSO regression on the TCGA training set (Figure 5A). In addition, multivariate Cox regression analyses confirmed that each selected ROG was an independent risk predictor for HGGs (Figure 5B). According to the regression coefficients of the 13 genes, we defined glioma-redox risk score (GRORS) as following: 0.192 × MTHFS + 0.151 × ALDH3B1 + 0.076 × GGT5 + 0.070 × DHFR + 0.035 × FKBP1B + 0.034 × ADH5 + 0.029 × NCF2 + 0.016 × GSTK1 + 0.009 × CASP3 + 0.008 × GPI + 0.006 × PDIA4 − 0.010 × MTHFD2 − 0.014 × TXN2. With the median of GRORS as the cutoff, we put the patients into the high-risk group and the low-risk group. Intriguingly, the OS of the high-risk group was much worse than the other group in both the training set and validation set of TCGA (Figures 5C, D). We also found that the survival outcomes based on CGGA and WCH sets were in line with the TCGA cohort (Figures 5E, F). Both univariate and multivariate Cox regression indicated that GRORS was an independent risk predictor for HGGs (Figures 5G, H).

### The risk model for prognostic prediction of HGGs

**FIGURE 6:**
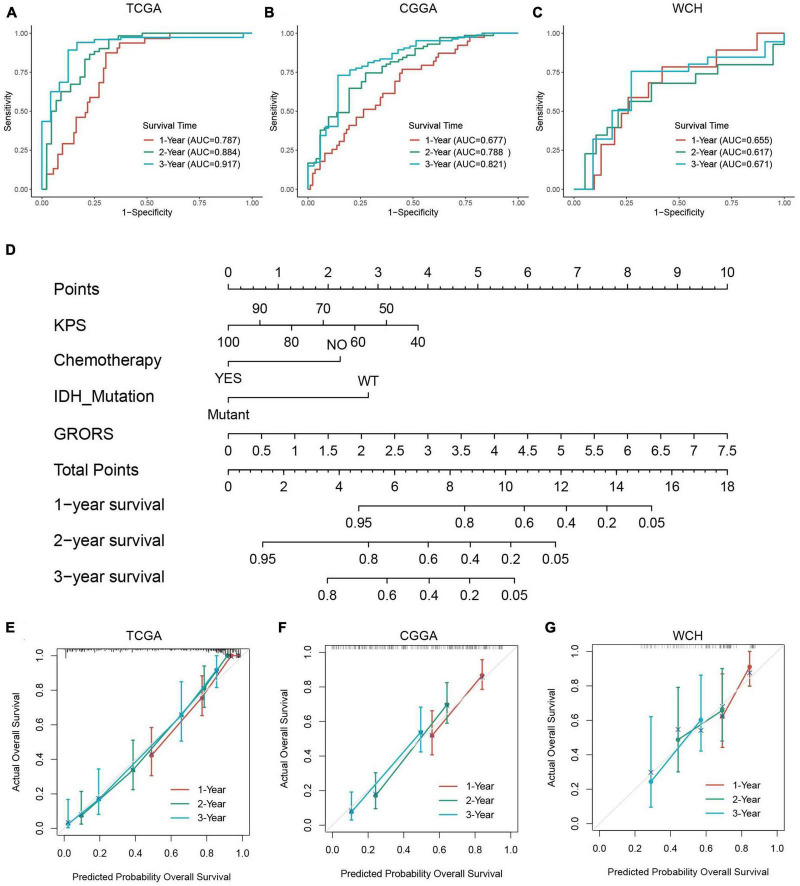
Capacity of prognosis prediction of GRORS and nomogram with its assessment. **(A–C)** ROC curves for predicting 1–3-year OS in TCGA cohort **(A)**, CGGA cohort **(B)** and WCH cohort **(C)**. **(D)** Nomogram based on GRORS and independent prognostic clinical variables. **(E–G)** Calibration curves for assessing the predictive capacity of the nomogram in TCGA cohort **(E)**, CGGA cohort **(F)**, and WCH cohort **(G)**.

The ROC curve analysis was used to further examine the prognostic accuracy of GRORS. Our results showed that GRORS could effectively predict the OS for TCGA cohort, and the 3-year AUC was 0.917 (Figure 6A). For CGGA cohort and WCH cohort, the 3-year AUC was 0.821, and 0.671, respectively, (Figures 6B, C). This meant that GRORS had a satisfactory performance in the prediction of HGG patients’ clinical outcomes. Nomogram is usually used to quantitatively estimate clinically individual risk by integrating a series of factors. Herein, we established a nomogram based on the variables with adjusted *P*-values < 0.05 in the multivariate Cox regression, assessing the prognosis of HGG patients in the 1–3 years. The calculated C-index was 0.835 of the TCGA cohort, compared to 0.796 for nomogram of GRORS only (Figure 6D). For the CGGA and the WCH cohort, C-indexes for all variables and GRORS only was 0.717, 0.687 (Supplementary Figure 5A), and 0.616, 0.626, respectively, (Supplementary Figure 5B). Furthermore, we compared prognostic accuracy of 3-year survival of several predictors with GRORS, results showing that GRORS was the leading predictor among all factors in the three cohorts, respectively, (Supplementary Figures 5C–E). The calibration plots of these cohorts indicated that the predictive survival rate of nomograms was consistently in accordance with the actual survival rate (Figures 6E–G).

### Biological, clinical, and immune characteristics between two risk groups

**FIGURE 7:**
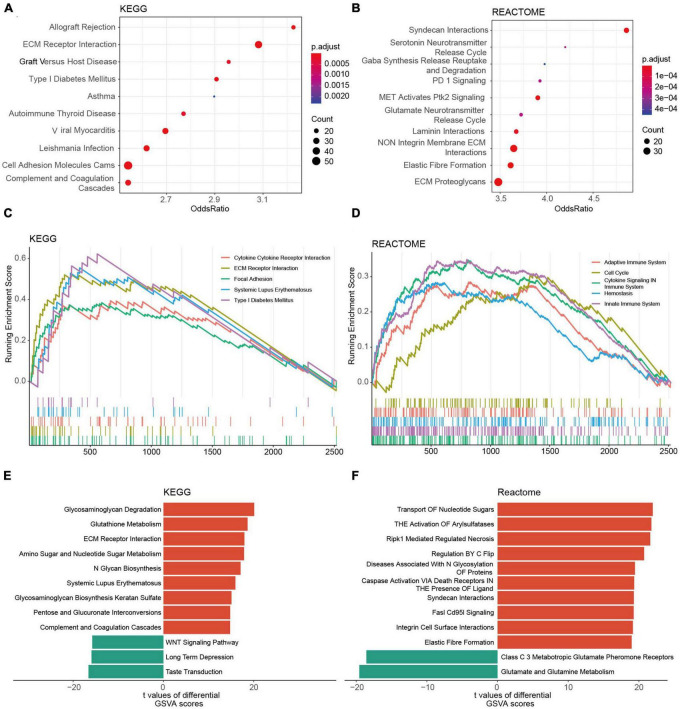
Functional annotations of DEGs between the risk groups based on the TCGA cohort. **(A,B)** Top 10 KEGG pathways **(A)** and REACTOME pathways **(B)** of the DEGs. **(C,D)** Top 5 KEGG pathways **(C)** and REACTOME pathways **(D)** of GSEA. **(E,F)** Top 10 KEGG pathways **(E)** and REACTOME pathways **(F)** of GSVA.

The KEGG analysis revealed that the genes of the two risk groups were associated with immune-related events, such as allograft rejection, complement and coagulation cascades (Figure 7A and Supplementary Figure 6A). The analysis based on REACTOME indicated that these genes were mainly enriched in neurotransmission and PD-1 signaling (Figure 7B and Supplementary Figure 6B). We also conducted GSEA analysis to study the distinctions of biological functions between the two risk groups. Results revealed that the DEGs of high-risk group were mainly associated with cytokine-cytokine receptor interaction (NES = 2.197), ECM receptor interaction (NES = 2.833), focal adhesion (NES = 2.463), and SLE (NES = 2.818) in terms of KEGG, respectively, (Figure 7C). Meanwhile, it was the adaptive immune system (NES = 2.149), cell cycle (NES = 2.212), cytokine signaling in the immune system (NES = 2.785), and innate immune system (NES = 2.871) that were enriched in DEGs of cluster 1 according to REACTOME (Figure 7D). Furthermore, the result of GSVA backed the results above as well (Figures 7E, F). We also found that a high GRORS was mainly enriched in HGG patients with higher WHO grade, IDH wildtype, 1p19q non-codeletion, ATRX wildtype, MGMT promoter un-methylated, TERT promoter mutant, and more malignant histology (Supplementary Figures 7A–I).

**FIGURE 8:**
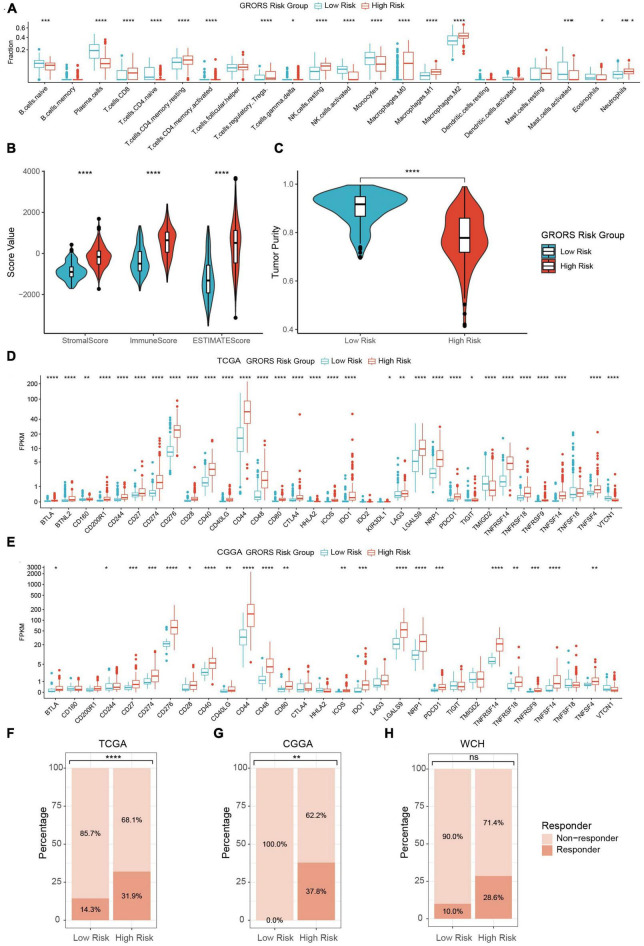
Immune phenotypes of two risk group. **(A)** Estimated fraction of 22 tumor infiltrating immune cells with CIBERSORTx algorithm. **(B)** Stromal, immune and ESTIMATE score with the method of ESTIMATE. **(C)** Tumor purity estimation with the method of ESTIMATE. **(D)** mRNA expression of 33 tumor infiltrating immune cells in TCGA cohort. **(E)** mRNA expression of 30 ICPs in CGGA cohort. **(F–H)** Estimation of potential therapeutic response to ICBs in TCGA **(F)**, CGGA **(G)**, and WCH cohort **(H)**. *p < 0.05, **p < 0.01, ***p < 0.001, ****p < 0.0001.

In our study, the proportion of activated NK cells, monocytes, and plasma cells in the high-risk group was prominently higher than in the low-risk group. Contrarily, the immunosuppressive cells, such as regulatory T cells (Tregs), tumor-associated macrophages, and neutrophils were found to be enriched in the high-risk group (Figure 8 and Supplementary Figures 8A, B). The ESTIMATE analysis demonstrated that the stromal score, immune score, and ESTIMATE score of the high-risk group were significantly higher than those of the other group. Besides, the tumor purity of the high-risk group was lower than its counterpart (Figures 8B, C and Supplementary Figures 8C–F). On the other hand, RNA-seq data revealed that a variety of immune deficiency markers, such as CD274 (PD-L1), CTLA4, NRP1, and LAGLS9, were increasingly expressed in the high-risk group, which was in line with our previous findings as regards immune suppression of cluster 1 determined by ROGs (Figures 8D, E and Supplementary Figure 8G). In the TIDE analysis, we found that HGG patients in the high-risk group exhibited higher potential sensitivity to ICBs compared to the low-risk groups (Figures 8F–H). Taken together, our findings suggest that HGG patients with high GRORS should be eligible for immunotherapy.

## Discussion

High-grade gliomas are a kind of highly fatal tumors accounting for about 50% of gliomas (Stupp et al., 2009; Ostrom et al., 2021). Although adopting standard surgical resection, radiotherapy plus concomitant chemotherapy, and chemotherapy, the therapeutic effect remains to be improved urgently (Chen et al., 2017). Among countless tumor-promoting factors, the altered metabolism of tumor cells is a main contributor to high malignancy for HGGs (Bi et al., 2020). New insights into the effect of Redox homeostasis on the vicious evolution of HGGs are rapidly emerging. GBM cells can oxidize glucose through glycolysis to supply other biosynthetic activities, for example, forming a large glutamine pool in the tumors, which is critical to glutathione biosynthesis and to promote aggressive tumor growth (Marin-Valencia et al., 2012). Given the protective effect of GSH for radiation and oxidation stress cytotoxicity, research finds that 2-oxoglutarate(2-OG)dependent transaminases, branched-chain amino acids transaminases (BACT1/2), and glutaminase exert complementary roles in GSH biosynthesis. The overproduction of 2- hydroxyglutarate (2-HG) in IDH mutant gliomas potently inhibits BACT1/2, which explains why the glutaminase inhibitors specifically sensitized IDH mutant gliomas to oxidation stress compared to IDH wild-type gliomas and suggest the strategy on the basis of redox to obtain maximum effectiveness for gliomas should take into consideration of comprehensive GSH biosynthesis pathways (McBrayer et al., 2018). Moreover, the activated AKT/NRF2/HO-1 oxidative stress axis confers amplified defense against ROS. Eventually, it causes glioblastoma resistant to TMZ treatment, which suggests redox imbalance is an important regulator of resistance to gliomas (Chuang et al., 2021). Our study further investigated the association between ROGs and the survival of HGG patients by taking advantage of bioinformatics. We also constructed a prognostic model based on these genes to predict the clinical outcomes and immunotherapy response of HGGs.

Cancer cells are usually characterized by imbalanced redox status owing to high levels of oxidative stress mainly exerted by ROS. Notably, the disturbance of redox could affect the genesis and development of tumors from varied aspects, for example, reshaping immune landscape of the TME (Gorrini et al., 2013; Kennel and Greten, 2021). The altered redox balance in ovarian cancers significantly elevates the ROS level, which favors tumor growth by leading to a reduction of macrophage migration and decreasing of CD8^+^ T-lymphocyte through PD-L1 upregulation (Li et al., 2022). It suggests that ROS plays a vital role in tumor immunosuppression formation. We divided the HGG patients into two clusters according to the ROG levels. We found that the patients of cluster 1 had a worse OS than cluster 2. The DEGs of two clusters were enriched in the processes of immune response and signaling. In addition, other immune-associated diseases, such as T1DM (Ilonen et al., 2019) and SLE (Thanou et al., 2021) are also related to the DEGs, which endorse the interaction between redox and immunity. It was reported that the interplay of these immune systems could regulate redox production, and vice versa in numerous diseases including tumors and inflammatory diseases (Sun et al., 2020). IDH mutation can affect mitochondria biochemistry of tumor cells by enhancing the enzymatic activity (Hvinden et al., 2021), then inducing an alteration of the redox status. Intriguingly, we found that the redox subtype of cluster 2 is significantly associated with IDH mutation in HGGs.

Our study suggests that ROGs of HGGs are associated with immune and metabolism of tumors. In order to further understand the effect of ROGs on the immune reactivity of HGGs, we find that HGGs of cluster 1 contain more tumor-promoting immune cells, suggesting that the redox status of cluster 1 liable to generate an immunosuppressive TME. A previous study reported that a hypoxia TME could trigger immunosuppression through hypoxia-inducible factor (HIF), with both M1 and M2 macrophages’ participation (Grabowski et al., 2021). This may partly explain why both M1 and M2 macrophages are increasing in HGGs of cluster 1. Glutaredoxin regulates redox homeostasis in many cancers. Its coding gene GLRX is an independent prognostic factor in glioma, and closely associates with an immunosuppressive tumor microenvironment with GLRX being precisely expressed in the M0 macrophages (Chang et al., 2020). *In vivo* investigations will need to determine the effect of each macrophage subtype on the immunosuppression of HGGs mediated by ROGs.

We next established a scoring system based on 13 ROGs, GRORS, which was proved to be an independent risk predictor for HGGs. Among genes of GRORS, most of them were antioxidative genes (Marchitti et al., 2010; Raza, 2011; Wickham et al., 2011; Wang et al., 2020). Our work also illuminated an interaction between GRORS and the tumor immune profiles. We found that HGGs with high GRORS contains a high fraction of the immunosuppressive cells, such as regulatory T-cells (Tregs), tumor-associated macrophages, and neutrophils. Tregs can inhibit antigen-presenting cells by CTLA-4, consuming IL-2, and producing immune inhibitory cytokines and molecules (Togashi et al., 2019). Tregs exert an immunosuppressive function by inhibiting CD4^+^ effector T-cells with a mechanism of inducing redox perturbation, including decreased GSH biosynthesis (Yan et al., 2010). After switching to an M2-like state, TAMs promote angiogenesis by secreting pro-angiogenic factors, suppressing T-cell infiltration and cytotoxic T-cell function, and remodeling ECM (Myers et al., 2019). Nuclear Factor (erythroid-derived 2)-like 2 (NrF2) is a vital regulator keeping the homeostasis of oxidative stress through initiating anti-oxidative genes expression (Kensler et al., 2007). Cancer cell-produced lactate can activate Nrf2 of macrophages which leads to macrophage polarization toward an M2-like phenotype. In turn, the M2-like macrophage upregulates Nrf2 expression of cancer cells to promote the epithelial-mesenchymal transition of tumors (Feng et al., 2018). Tumor-associated neutrophils are capable of suppressing innate and adaptive lymphoid cell function by producing ROS, reactive nitrogen intermediates (RNI), ARG1 (Jaillon et al., 2020). The GSH system of the activated neutrophils affects function of these cells heavily by stimulation of glutathione reductase. Moreover, cellular redox status can significantly influence the function of individual neutrophils. For example, persistently upregulated ROS may result in the internalization of membrane chemokine receptors, CXCR2, thus suppressing neutrophil migration (Morris et al., 2022). In total, redox can interact with multiple components in tumors and regulate the aggressive growth of tumors.

Similarly, immune deficiency markers, such as CD274 (PD-L1), CTLA4, NRP1, and LAGLS9, were increasingly expressed in the high GRORS tumors. The representative immune checkpoints of PD-L1/PD-1 and CTLA4/B7 1/2 can potently block the generation of stimulating cytokines, such as IFN-γ, tumor necrosis factor-α (TNF-α), and IL-2, which decreases the immunoreactivity of tumors (Kiaie et al., 2021). Galectin-9 (LGALS9) can induce T cell death through binding with TIM-3 (Yang et al., 2021). Moreover, CD48 could act as an immunosuppressive mediator by enhancing the function of Tregs in hepatocellular carcinomas (Wang et al., 2021). Similarly, IDO1 promotes immune escape in multiple tumors such as melanoma, colon cancer, and glioma by regulating Tregs (Zhai et al., 2015; Munn and Mellor, 2016). Therefore, HGGs with high GRORS have the characteristic of antioxidative ability and an immunosuppressive TME and thus suffering a poor outcome.

Immunotherapy is a promising treatment for many cancers, which part of mechanisms related to redox. PD-1 inhibition promotes tumor cell ferroptosis through interferon-gamma (Wang et al., 2019). Unfortunately, current immunotherapies on gliomas have been all far from effective (Sener et al., 2022). Glutathione peroxidase 2 (GPX2), a member of GPX family (GPX1-8), protects cells against from oxidative damage by exhausting a wide range of ROS using GSH. Apart from scarce pan-leukocyte infiltration in immunologically cold tumors, the metabolic enzyme of GPX2 overexpressed in several smoking-related cold tumors is another novel targetable effector mediating tumor immune escape. Tumors with GPX2 overexpression have a more incompetent tumor immune environment (Ahmed et al., 2022). The TME can influence the efficacy of cancer therapy and is currently considered another therapeutic target (Quail and Joyce, 2017). These suggest that alteration of immune landscape of HGGs may be a possible way to improve the efficacy of immunotherapy. A study has reported that inhibition of SIRT6/NF-κB by icariin can alter redox status and enhance anti-tumor immunity and thus contain the growth of triple-negative breast cancer (Song et al., 2020), indicating that the immune reprogramming strategy could be performed through redox intervention. Our results indicate high sensitivity to ICBs of HGGs with high GRORS, suggesting future studies should try to adopt ICBs to overcome these fatal HGGs. On the other hand, immune reprogramming should be investigated to increase the response of HGGs with low GRORS for ICBs.

There are inevitably a handful of limitations in this study. Firstly, this study focuses on gliomas of WHO grades 3 and 4, precluding the low-grade gliomas. Instead of experimental validation, the analysis of immune cells is estimated by algorithms that are not so precise enough that avoid biases. In addition, our study doesn’t explore the mechanism of ROG regulating redox balance and further exhaustive experiments are required. Finally, this study uses retrospective data, meaning that a prospective cohort study is needed for further validation.

In summary, our study points toward the redox-related gene signature playing an important role in predicting prognosis and reshaping the immune features of TME of HGGs and highlights GRORS is a promising predictor for the therapeutic response of ICBs to HGGs.

## Data availability statement

The datasets presented in this study can be found in online repositories. The names of the repository/repositories and accession number(s) can be found in the article/Supplementary material.

## Ethics statement

The studies involving human participants were reviewed and approved by the Institutional Review Board of West China Hospital. The patients/participants provided their written informed consent to participate in this study.

## Author contributions

YY, MZ, YL, and MC had the original ideas of this manuscript. SC, YY, WF, and ZW retrieved the data from the databases. SZ, MZ, and YY performed the statistical analysis. MZ and SZ interpreted the results. All authors read and approved this manuscript.

## Footnotes

1
https://www.cancer.gov/tcga

2
http://www.cgga.org.cn

3
https://www.gsea-msigdb.org

4
https://cibersortx.stanford.edu/

## Supplementary material

Supplementary Figure 1 Consensus clustering of IDH-mutant (IDHmut) and IDH-wildtype (IDHwt) HGGs. **(A)** Consensus index matrix of IDHmut HGGs with ROGs when the number of cluster (k) were 3. **(B)** Cumulative distribution functions (CDFs) of consensus index when k was set from 2 to 10 in IDHmut HGGs. **(C)** Gain of area under the CDFs when k was set from 2 to 10 in IDHmut HGGs. **(D)** Consensus index matrix of IDHwt HGGs with ROGs when (k) were 3. **(E)** CDFs of consensus index when k was set from 2 to 10 in IDHwt HGGs. **(F)** Gain of area under the CDFs when k was set from 2 to 10 in IDHwt HGGs.

Supplementary Figure 2 Transcriptome profiles of redox subclusters in the IDH-mutant (IDHmut) HGGs. **(A–C)** The KEGG, REACTOME, and msigH functional enrichment of differentially expressed genes between subcluster 1/2 and subcluster 3 in the IDHmut HGG, respectively. Only pathways with gene counts over 10 were plotted. Right panel enriched pathways with top 10 odds ratio.

Supplementary Figure 3 Transcriptome profiles of redox subclusters in the IDH-wildtype (IDHwt) HGGs. **(A–C)** The KEGG, REACTOME, and msigH functional enrichment of differentially expressed genes between subcluster 1/2 and subcluster 3 in the IDHmut HGG, respectively. Only pathways with gene counts over 10 were plotted. Right panel enriched pathways with top 10 odds ratio.

Supplementary Figure 4 Status of immune biomarkers of redox subclusters in IDH-mutant (IDHmut) and IDH-wildtype (IDHwt) HGGs. **(A)** mRNA expression of immune biomarkers in IDH-mutant (IDHmut) and IDH-wildtype (IDHwt) HGGs. ns, not significant, **p* < 0.05, ^**^*p* < 0.01, ^***^*p* < 0.001, ^****^*p* < 0.0001.

Supplementary Figure 5 Nomogram based on CGGA and WCH cohort and predicting performance of different variables on 3-year OS. **(A,B)** Nomogram of HGGs based on CGGA cohort **(A)** and WCH cohort **(B)**. **(C–E)** ROC curves for different variables predicting 3-year OS in TCGA cohort **(C)**, CGGA cohort **(D)** and WCH cohort **(E)**.

Supplementary Figure 6 Heatmaps of top 20 differentially expressed gene sets with different GRORS. **(A)** Heatmap with KEGG. **(B)** Heatmap with REACTOME dataset.

Supplementary Figure 7 The relation between GRORS and clinic-pathological indicators. The indicator included gender **(A)**, WHO grade **(B)**, IDH status **(C)**, 1p19q codeletion **(D)**, ATRX status **(E)**, MGMT promoter status **(F)**, TERT promoter status **(G)**, and histology **(H)**. **(I)** The heatmap of clinic-pathological indicators distribution according to the GRORS, and distribution of genes included in GRORS calculation. ns, not significant; ^****^*p* < 0.0001.

Supplementary Figure 8 Immune phenotypes of two risk groups in CGGA and WCH cohort. **(A,B)** Fraction of 22 infiltrating immune cells with CIBERSORTx algorithm in CGGA cohort **(A)** and WCH cohort **(B)**. **(C,E)** Stromal, immune and ESTIMATE score with the method of ESTIMATE in CGGA cohort **(C)** and WCH cohort **(E)**. **(D,F)** Tumor purity estimation with the ESTIMATE and CPE algorithms in CGGA cohort **(D)** and WCH cohort **(F)**. **(G)** mRNA expression of 33 I in WCH cohort. **p* < 0.05, ^**^*p* < 0.01, ^***^*p* < 0.001, ^****^*p* < 0.0001.
