# Supplementary material for: Impact of redox-related genes on tumor microenvironment immune characteristics and prognosis of high-grade gliomas
Source: Front Cell Neurosci. 2023 May 12;17:1155982. doi: 10.3389/fncel.2023.1155982 (PMC10213429; doi:10.3389/fncel.2023.1155982)
Supplement: Supplementary file 1 [file Table_1.DOCX]

Supplementary Table S1. Clinic-pathological characteristics of HGG patients in TCGA, CCGA, and WCH cohort.

|  | **TCGA** | **CGGA** | **WCH** |
| --- | --- | --- | --- |
| Total Tumor | 391 | 132 | 48 |
| Normal Brain | 5 | - | 16 |
| Age | 53 (21 - 89) | 50 (18 - 79) | 44 (19 - 77) |
| Gender |  |  |  |
| Female | 155 | 50 | 20 |
| Male | 236 | 82 | 28 |
| NA | 0 | 0 | 0 |
| Histology |  |  |  |
| Astrocytoma | 167 | 36 | 6 |
| Oligodendroglioma | 70 | 12 | 8 |
| Glioblastoma | 154 | 84 | 34 |
| NA | 0 | 0 | 0 |
| Grade |  |  |  |
| G3 | 237 | 48 | 14 |
| G4 | 154 | 84 | 34 |
| NA | 0 | 0 | 0 |
| IDH Status |  |  |  |
| Mutant | 181 | 32 | 20 |
| WT | 208 | 100 | 28 |
| NA | 2 | 0 | 0 |
| 1p19q Codeletion |  |  |  |
| Codel | 70 | 10 | 7 |
| Non-codel | 314 | 120 | 31 |
| NA | 7 | 2 | 10 |
| TERT Promoter Status |  | - |  |
| Mutant | 225 | - | 24 |
| WT | 75 | - | 18 |
| NA | 91 | - | 6 |
| MGMT Promoter Methylation |  |  |  |
| Methylated | 242 | 55 | 25 |
| Unmethylated | 116 | 71 | 12 |
| NA | 33 | 6 | 11 |
| ATRX Status |  | - |  |
| Mutant | 83 | - | 15 |
| WT | 300 | - | 31 |
| NA | 8 | - | 2 |

Abbreviation: HGG, high-grade glioma; TCGA, The Cancer Genome Atlas; CGGA, Chinese Glioma Genome Atlas; WCH, West China Hospital; IDH, isocitrate dehydrogenase; TERT, telomerase reverse transcriptase; MGMT, O6-methylguanine-DNA methyltransferase; ATRX, Alpha Thalassemia/Mental Retardation, X-linked; WT, wild type; NA, not available.
