# Supplementary material for: Impact of redox-related genes on tumor microenvironment immune characteristics and prognosis of high-grade gliomas
Source: Front Cell Neurosci. 2023 May 12;17:1155982. doi: 10.3389/fncel.2023.1155982 (PMC10213429; doi:10.3389/fncel.2023.1155982)
Supplement: Supplementary file 2 [file Table_2.DOCX]

Supplementary Table S2. The result of retrieve of ROGs on MSigDB.

| Pathway | Number of genes |
| --- | --- |
| Total (Unique Genes) | 204 |
| Directly Redox Related (Benfeitas et.al) | 116 |
| Cell Redox Homeostasis | 55 |
| Indirectly Redox Related (Benfeitas et.al) | 38 |
| Response to Redox State | 13 |
| Peroxiredoxin Activity | 7 |
| Peroxiredoxin 2 Induced Ovarian Failure | 7 |
| Thioredoxin Disulfide Reductase Activity | 6 |
| Thioredoxin Peroxidase Activity | 5 |
