# Supplementary figures and images for: Impact of redox-related genes on tumor microenvironment immune characteristics and prognosis of high-grade gliomas
Source: Front Cell Neurosci. 2023 May 12;17:1155982. doi: 10.3389/fncel.2023.1155982 (PMC10213429; doi:10.3389/fncel.2023.1155982)

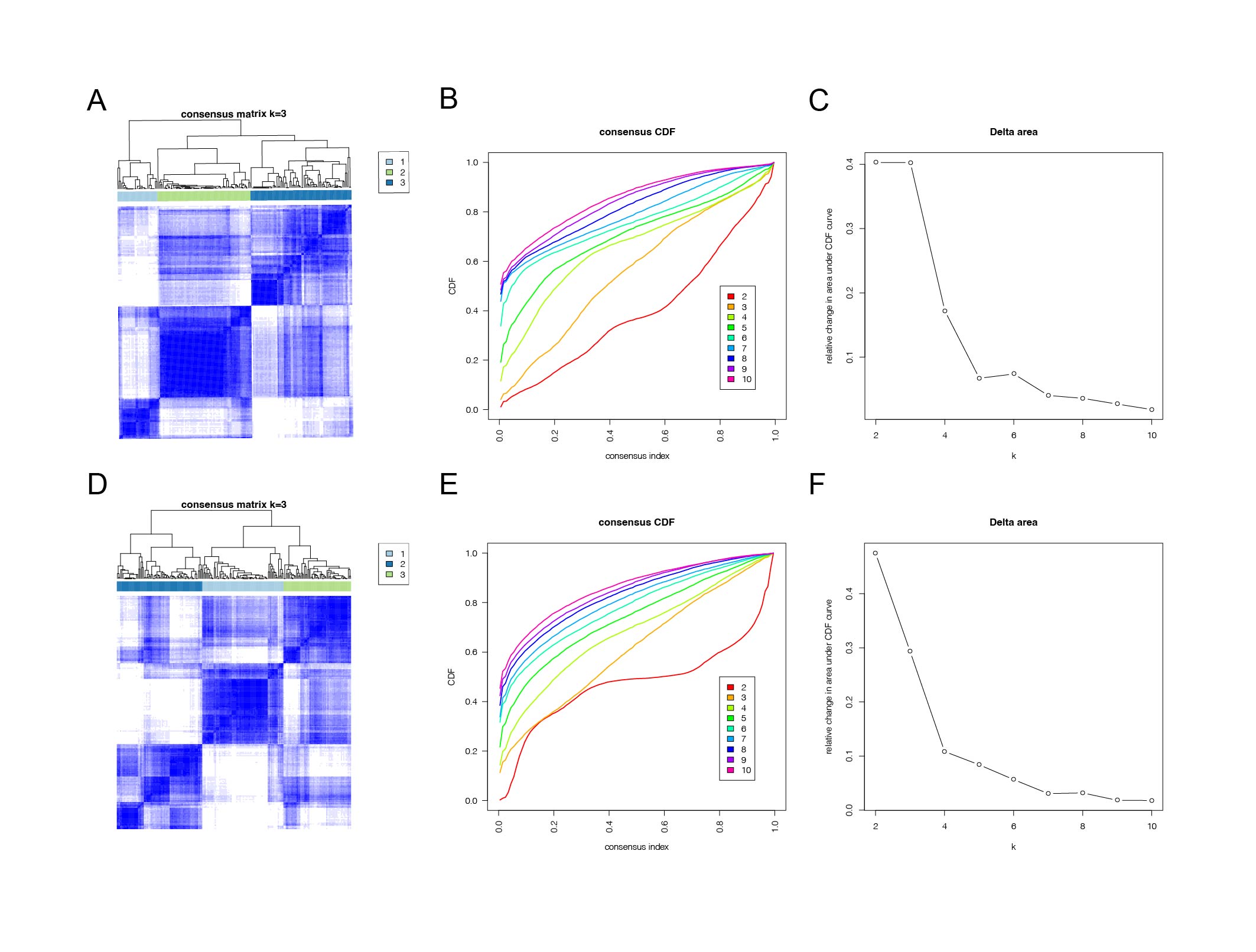

Supplement: Supplementary Figure 1 — Consensus clustering of IDH-mutant (IDHmut) and IDH-wildtype (IDHwt) HGGs. (A) Consensus index matrix of IDHmut HGGs with ROGs when the number of cluster (k) were 3. (B) Cumulative distribution functions (CDFs) of consensus index when k was set from 2 to 10 in IDHmut HGGs. (C) Gain of area under the CDFs when k was set from 2 to 10 in IDHmut HGGs. (D) Consensus index matrix of IDHwt HGGs with ROGs when (k) were 3. (E) CDFs of consensus index when k was set from 2 to 10 in IDHwt HGGs. (F) Gain of area under the CDFs when k was set from 2 to 10 in IDHwt HGGs. [file Image_1.JPEG]

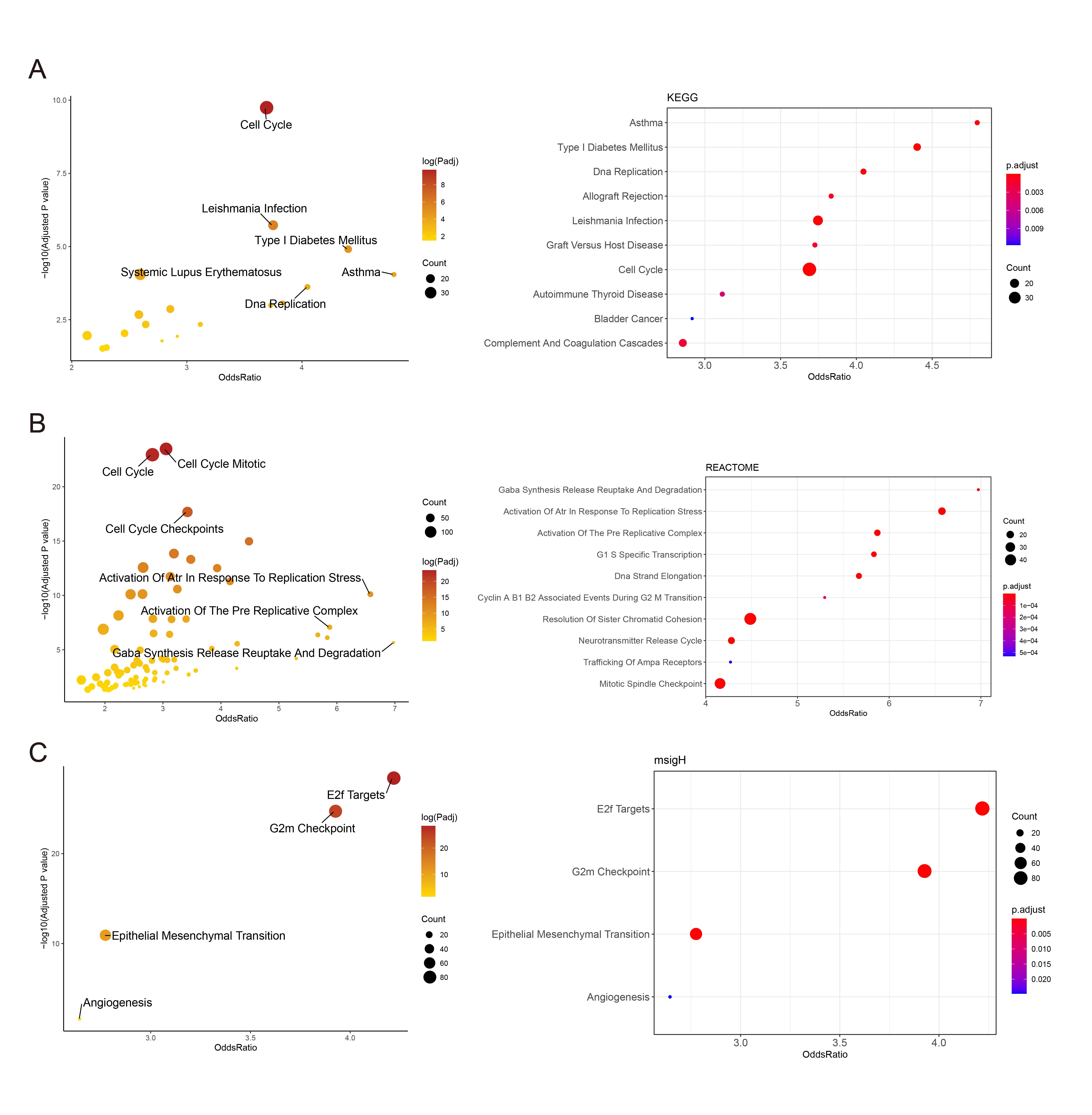

Supplement: Supplementary Figure 2 — Transcriptome profiles of redox subclusters in the IDH-mutant (IDHmut) HGGs. (A–C) The KEGG, REACTOME, and msigH functional enrichment of differentially expressed genes between subcluster 1/2 and subcluster 3 in the IDHmut HGG, respectively. Only pathways with gene counts over 10 were plotted. Right panel enriched pathways with top 10 odds ratio. [file Image_2.JPEG]

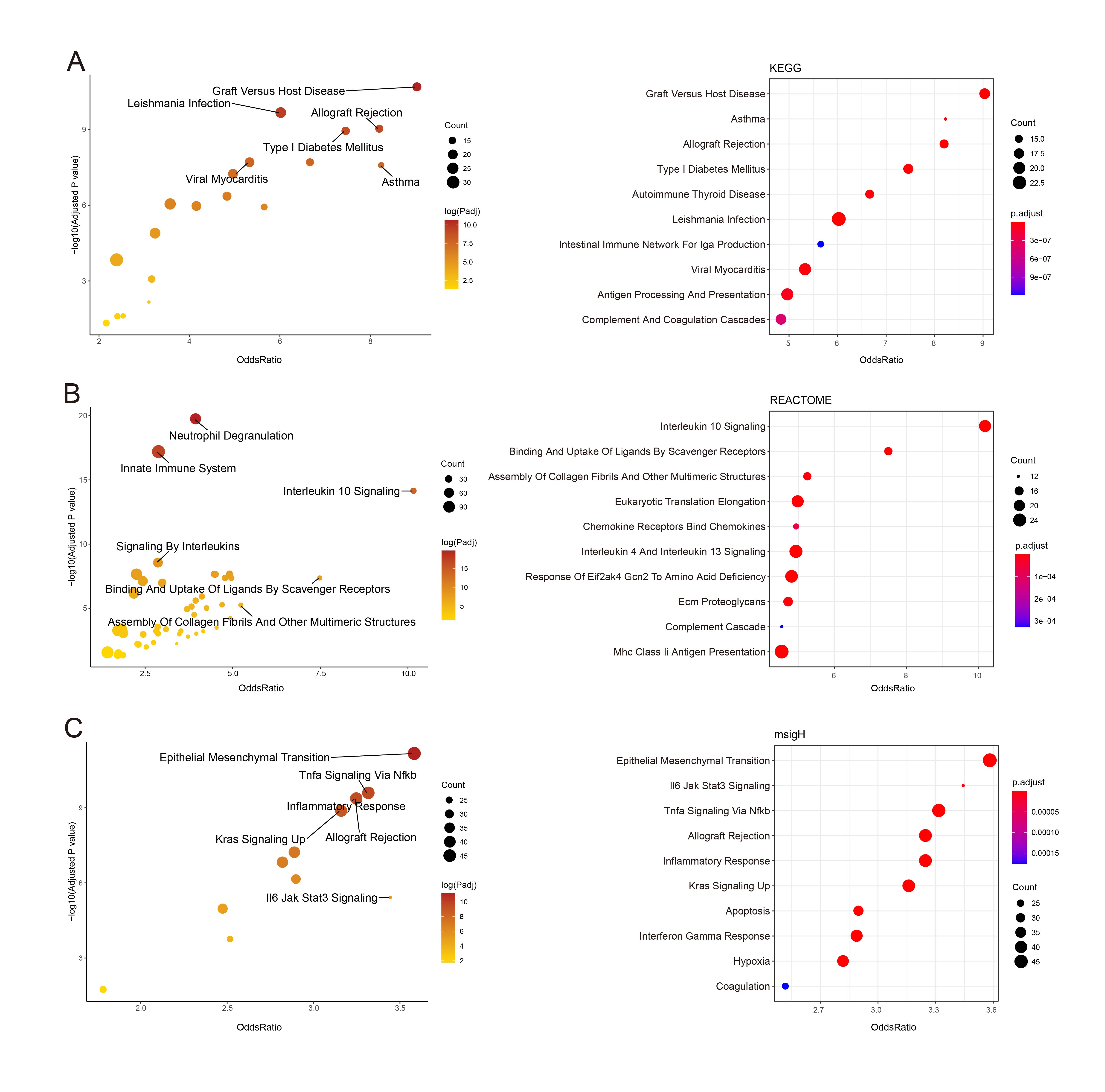

Supplement: Supplementary Figure 3 — Transcriptome profiles of redox subclusters in the IDH-wildtype (IDHwt) HGGs. (A–C) The KEGG, REACTOME, and msigH functional enrichment of differentially expressed genes between subcluster 1/2 and subcluster 3 in the IDHmut HGG, respectively. Only pathways with gene counts over 10 were plotted. Right panel enriched pathways with top 10 odds ratio. [file Image_3.JPEG]

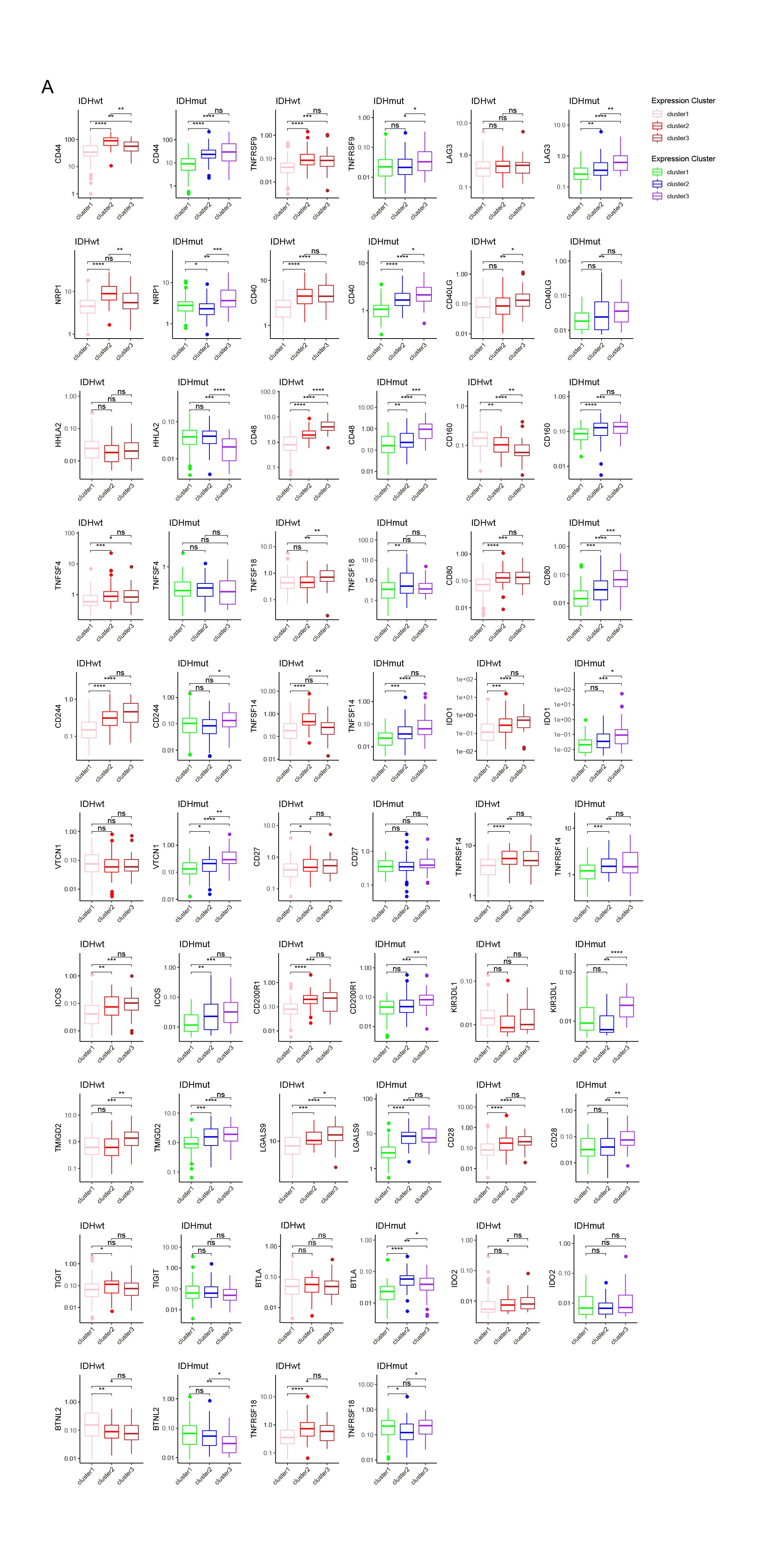

Supplement: Supplementary Figure 4 — Status of immune biomarkers of redox subclusters in IDH-mutant (IDHmut) and IDH-wildtype (IDHwt) HGGs. (A) mRNA expression of immune biomarkers in IDH-mutant (IDHmut) and IDH-wildtype (IDHwt) HGGs. ns, not significant, *p < 0.05, **p < 0.01, ***p < 0.001, ****p < 0.0001. [file Image_4.JPEG]

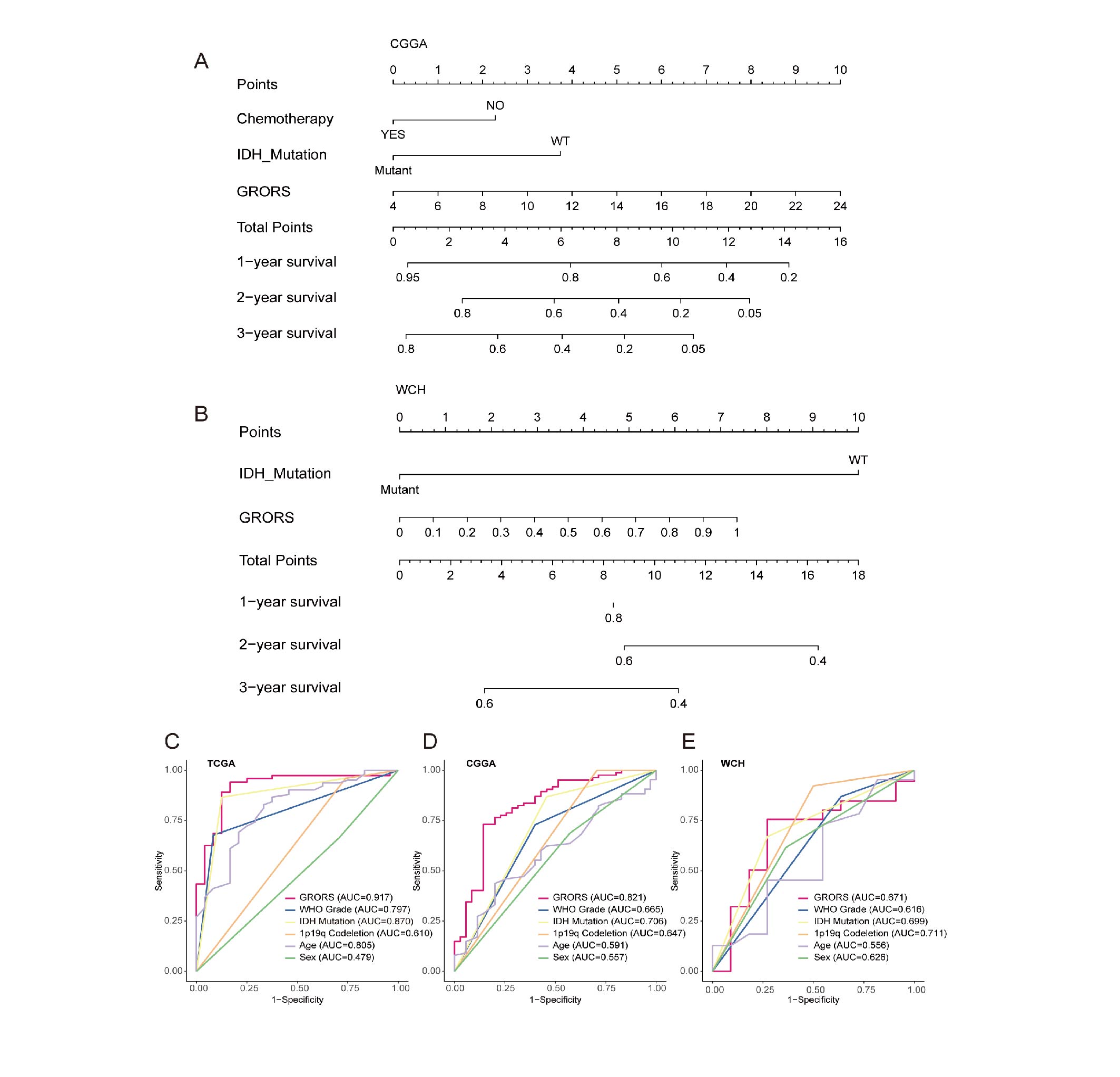

Supplement: Supplementary Figure 5 — Nomogram based on CGGA and WCH cohort and predicting performance of different variables on 3-year OS. (A,B) Nomogram of HGGs based on CGGA cohort (A) and WCH cohort (B). (C–E) ROC curves for different variables predicting 3-year OS in TCGA cohort (C), CGGA cohort (D) and WCH cohort (E). [file Image_5.JPEG]

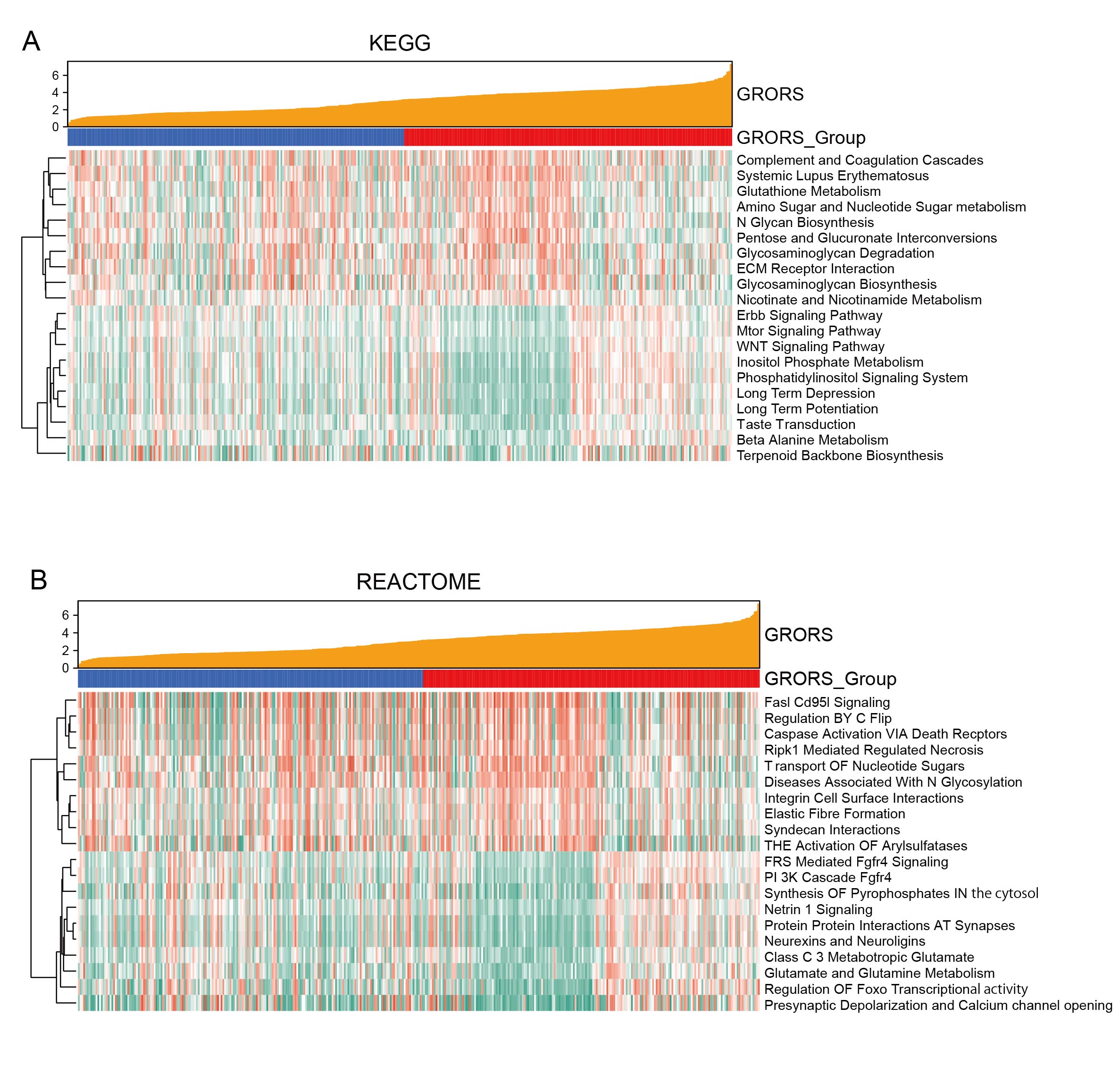

Supplement: Supplementary Figure 6 — Heatmaps of top 20 differentially expressed gene sets with different GRORS. (A) Heatmap with KEGG. (B) Heatmap with REACTOME dataset. [file Image_6.JPEG]

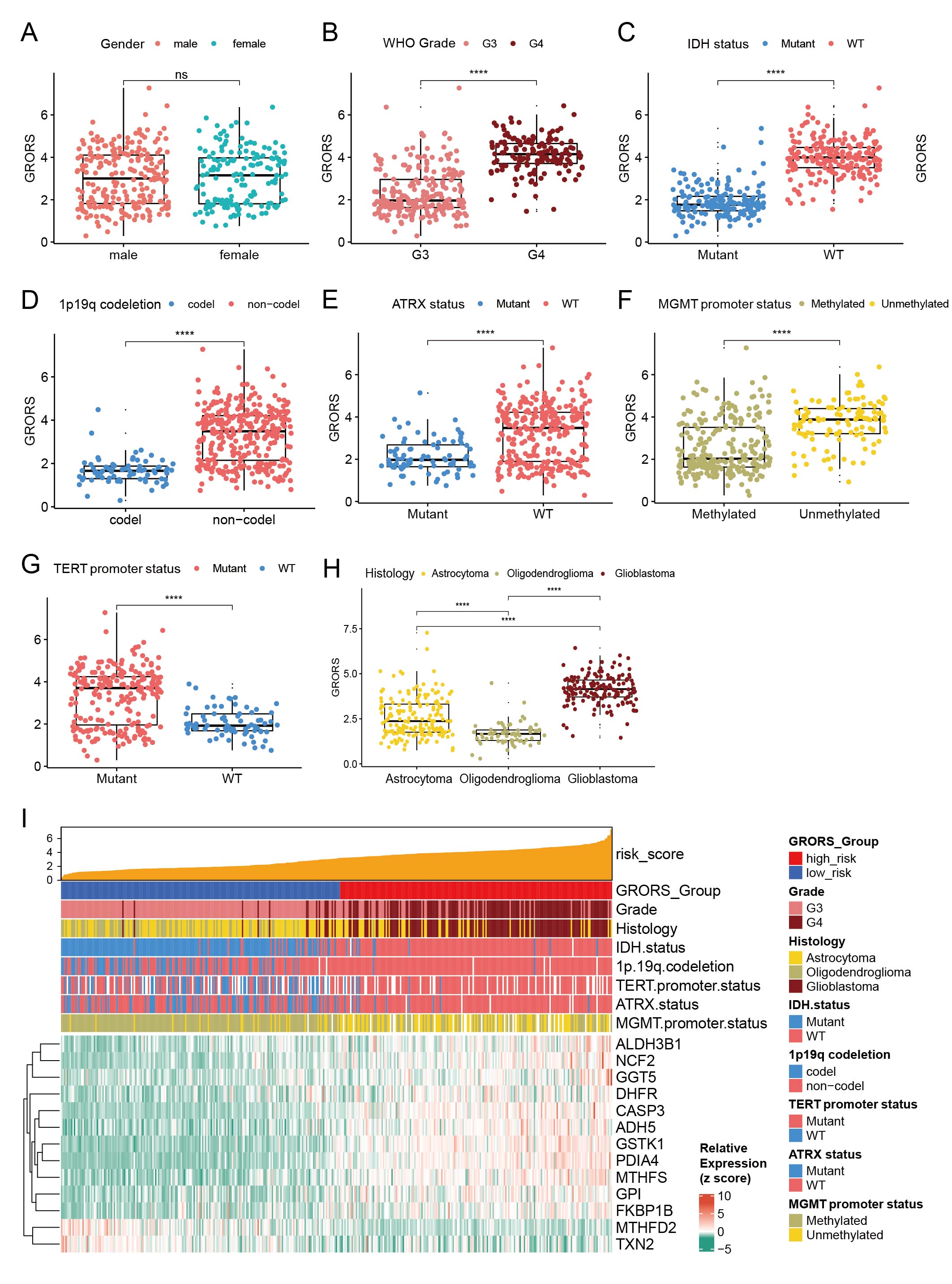

Supplement: Supplementary Figure 7 — The relation between GRORS and clinic-pathological indicators. The indicator included gender (A), WHO grade (B), IDH status (C), 1p19q codeletion (D), ATRX status (E), MGMT promoter status (F), TERT promoter status (G), and histology (H). (I) The heatmap of clinic-pathological indicators distribution according to the GRORS, and distribution of genes included in GRORS calculation. ns, not significant; ****p < 0.0001. [file Image_7.JPEG]

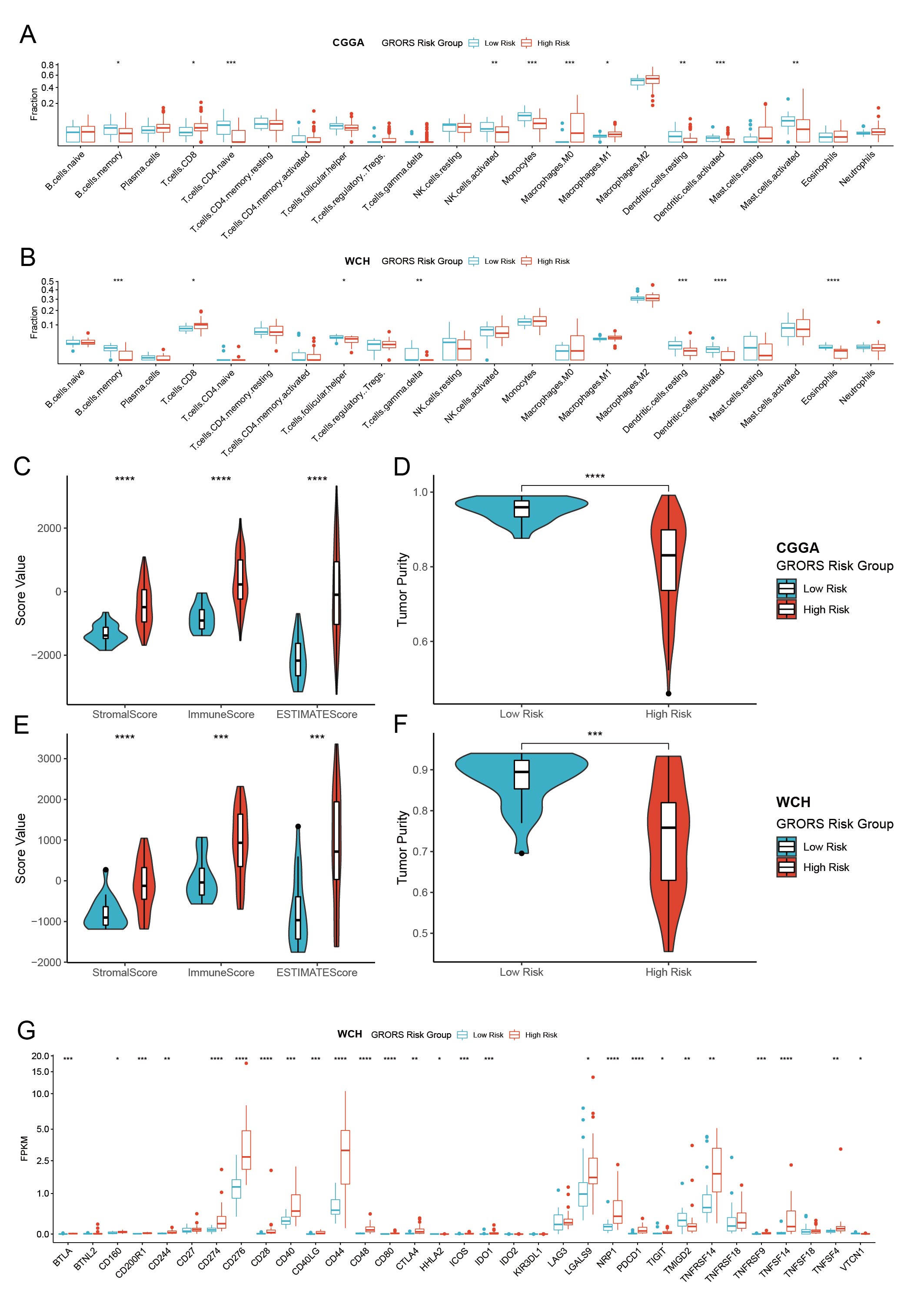

Supplement: Supplementary Figure 8 — Immune phenotypes of two risk groups in CGGA and WCH cohort. (A,B) Fraction of 22 infiltrating immune cells with CIBERSORTx algorithm in CGGA cohort (A) and WCH cohort (B). (C,E) Stromal, immune and ESTIMATE score with the method of ESTIMATE in CGGA cohort (C) and WCH cohort (E). (D,F) Tumor purity estimation with the ESTIMATE and CPE algorithms in CGGA cohort (D) and WCH cohort (F). (G) mRNA expression of 33 I in WCH cohort. *p < 0.05, **p < 0.01, ***p < 0.001, ****p < 0.0001. [file Image_8.JPEG]
